# Influence of Raw Material Fineness on Clinker Burnability and Cement Performance

**DOI:** 10.3390/ma18112453

**Published:** 2025-05-23

**Authors:** Shanshi Chen, Xinjian Yue, Yongmin Zhou

**Affiliations:** College of Materials Science and Engineering, Nanjing Tech University, South Puzhu Road No. 30, Nanjing 211816, China; 202261203311@njtech.edu.cn (S.C.); 202261203299@njtech.edu.cn (X.Y.)

**Keywords:** cement raw materials, particle size distribution, clinker sintering, cement performance

## Abstract

The particle size of raw materials is crucial for clinker formation, ultimately affecting cement performance. However, the specific effects of the fineness of individual raw materials on clinker burnability remain insufficiently understood. In this study, the fineness of limestone, shale, and iron-bearing materials was systematically varied to explore its influence on raw meal burnability and the resulting cement properties. Raw materials were prepared with controlled residue levels (5–20%) retained on an 80 μm sieve. Their impact was evaluated based on free lime content (f-CaO), clinker phase composition, cement strength development, and hydration behavior. Among the variables studied, limestone fineness was found to be the predominant factor affecting f-CaO levels, confirming its dominant role in governing clinker burnability. In contrast, fineness adjustments of aluminosilicate and iron-bearing components produced comparatively minor effects. Despite variations in raw meal fineness, clinkers produced with sieve residues between 10% and 15% exhibited consistent phase compositions, primarily comprising tricalcium silicate (C_3_S), dicalcium silicate (C_2_S), tricalcium aluminate (C_3_A), and tetracalcium aluminoferrite (C_4_AF). Furthermore, cement pastes derived from these clinkers demonstrated similar setting times, mechanical strengths, and hydration product assemblages. These results highlight the robustness of cement performance with respect to moderate variations in raw material fineness, particularly when limestone fineness is adequately controlled.

## 1. Introduction

Clinker formation is governed by a combination of raw meal chemistry [1,2], calcination temperature [3,4], and particle size distribution (PSD) [5], with the latter exerting a pronounced influence on solid-state reaction interfaces, diffusion pathways, and reaction kinetics [6]. Among these factors, PSD plays a critical role in determining the thermal behavior during sintering and the evolution of clinker phase assemblages.

Previous studies have identified an optimal specific surface area range of 250–320 m^2^/kg as favorable for the formation of major silicate phases, particularly C_3_S and C_2_S [7]. In contrast, excessively broad or ultra-fine PSDs can hinder the formation of a homogeneous liquid phase, reduce thermal efficiency during calcination, promote ring formation, and ultimately compromise both clinker quality and kiln operational stability [8,9,10]. Al-Salmany and Al-Hazaa [11] emphasized that particle size uniformity is vital for achieving stable sintering. When raw meal particles are refined below 80 μm, the resulting increase in surface area and reduction in diffusion distances enhance the reactivity between CaO and SiO_2_, promoting C_3_S formation and minimizing residual free lime (f-CaO) [12]. However, overly fine raw meals with specific surface areas exceeding 400 m^2^/kg may induce localized high-viscosity liquid phases, which obstruct effective heat transfer and impede reaction kinetics [13,14]. These effects destabilize the thermal regime and impair clinker formation. Consequently, optimization of raw meal PSD should prioritize both balance and uniformity, rather than indiscriminate fineness.

In addition to PSD, the alkali content of raw materials—though typically present in minor quantities—can influence the clinkerization process. Alkali oxides such as Na_2_O and K_2_O contribute to early liquid phase formation, affecting sintering temperature and phase evolution, particularly the development of C_3_A and C_4_AF [15]. Moreover, alkalis can lower the eutectic point of the melt, potentially interacting with PSD effects on reaction kinetics. While this study focuses primarily on PSD, alkali contents were controlled during raw meal preparation to isolate the influence of particle fineness from compositional variability [16].

Grinding and blending strategies also significantly affect clinker reactivity and cement performance. Garces-Vargas et al. [17] reported that non-optimized blending and grinding techniques may disrupt synergistic reactions among raw meal components, thereby limiting hydration activity and early strength development. Therefore, understanding the contribution of individual constituent fineness to overall reactivity is essential for improving clinker formation, enhancing energy efficiency, and reducing CO_2_ emissions [18,19].

Beyond clinker formation, raw material PSD directly influences the rheological properties and hydration kinetics of cementitious systems, which are critical in engineering applications such as grouting and soil stabilization. Prior research has shown that variations in cement fineness and composition significantly affect the time-dependent viscosity and flow characteristics of cement-based suspensions [20,21,22]. These findings underscore the broader relevance of raw material PSD control across the entire cement production and application spectrum.

In this study, the influence of raw material fineness on clinker burnability, mineral phase development, and cement performance is systematically investigated. Using controlled proportions of limestone, shale, and industrial by-products—namely iron tailings or copper slag—the effect of fineness variation was evaluated based on sieve residue values ranging from 5% to 20% at 80 μm. The assessment focused on f-CaO content, clinker mineralogy, and the compressive strength development of the produced cement, providing insight into the mechanistic role of individual raw material fineness in determining raw meal reactivity.

Unlike conventional approaches that regulate the overall fineness of the raw mix, this study independently adjusts the fineness of limestone, shale, and iron-bearing components. This approach aims to identify the dominant constituent influencing clinker burnability and cement strength, offering both empirical evidence and theoretical guidance for targeted PSD optimization. The findings hold significant implications for enhancing process efficiency, clinker quality, and sustainability in cement manufacturing.

Furthermore, the incorporation of industrial solid wastes such as copper slag and iron tailings into cement raw mixes has attracted increasing interest due to its potential to reduce natural resource consumption and environmental impacts. Rich in Fe_2_O_3_ and SiO_2_, these materials function as effective corrective components in clinker production while contributing to waste valorization and carbon footprint reduction. Their use aligns with global efforts toward sustainable construction and circular economy practices. Recent studies have further validated their viability—for example, copper slag as a supplementary raw material in clinker formulation [23], and iron tailings enhancing the performance and reducing the environmental load of blended cement systems [24].

## 2. Materials and Methods

### 2.1. Materials

**Figure 1:**
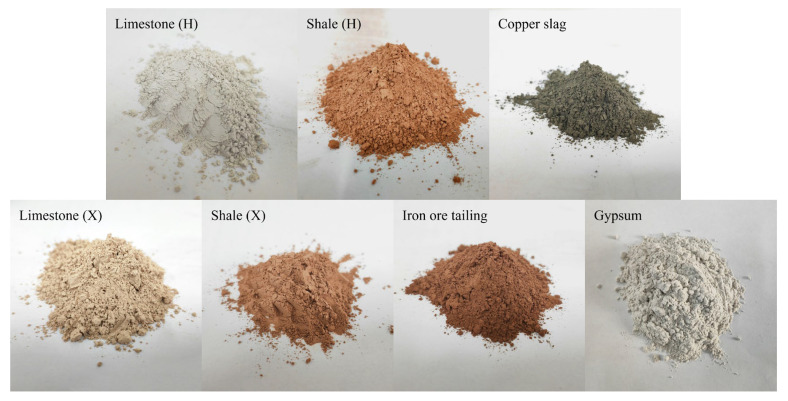
Raw materials used in this study.

As depicted in Figure 1, the raw materials for this study were sourced as follows: limestone (X), shale (X), and iron tailings from Zhejiang Xindu Cement Co. (Jiaxing, China), and limestone (H), shale (H), and copper slag from Hejiashan Cement Co. (Jiangshan, China). Gypsum, in the form of analytical reagent (AR)-grade calcium sulfate dihydrate, was provided by Xilong Chemical Co. (Shantou, China) In this study, iron tailings and copper slag were utilized as ferriferous corrective materials to effectively adjust the Fe_2_O_3_ content of the raw mix, ensuring it meets the requirements for clinker mineral formation. Their use also promotes the resource utilization of industrial solid wastes, contributing to reductions in both production costs and carbon emissions [25,26].

**Figure 2:**
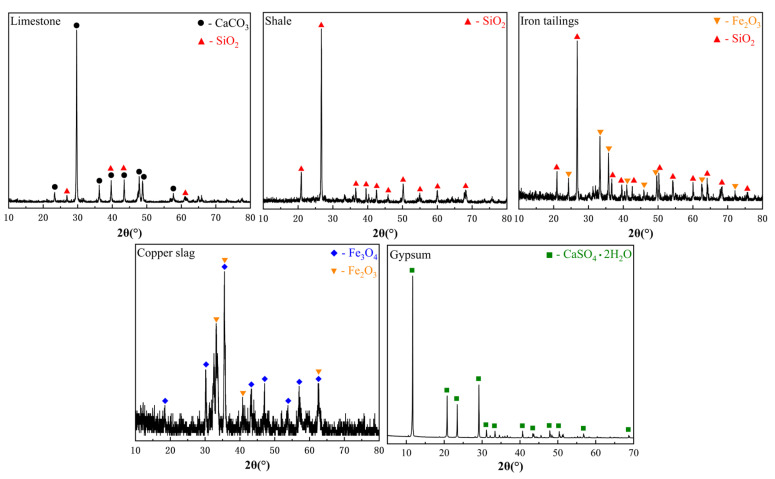
XRD patterns of various cement raw materials.

The chemical compositions of the milled raw materials were analyzed by X-ray fluorescence (XRF; Spectro, Bruker, Billerica, MA, USA), with the results summarized in Table 1. Mineralogical phases were identified through X-ray diffraction (XRD) using Cu Kα radiation, and the corresponding diffractograms are shown in Figure 2. Material densities were determined using a pycnometer and are reported in Table 2.

### 2.2. Methods

#### 2.2.1. Raw Meal Proportioning for Cement Production

Drawing on the chemical compositions presented in Table 1, two distinct raw meal formulations were developed through an optimization strategy to produce two types of Portland cement. The lime saturation factor (KH) was controlled at 0.9 ± 0.1, the silica modulus (SM) at 2.6 ± 0.1, and the alumina modulus (IM) at 1.6 ± 0.1 [27]. During the proportioning process, two of the three targeted modulus values were strictly maintained, while the third was adjusted as closely as possible to ensure a well-balanced raw mix with favorable burnability. The final raw meal compositions and corresponding modulus values of the resulting clinkers are presented in Table 3.

#### 2.2.2. Preparation of Raw Material Powder Samples with Varying Fineness

Raw materials were ground using appropriate techniques to achieve the target fineness levels, as specified in Table 4. Each material was milled separately to attain 80 μm sieve residues of 5%, 10%, 15%, and 20% (hereafter, all residue values refer to the 80 μm sieve). This fineness range was selected based on typical fineness control practices in cement plants and preliminary trials. This range effectively captures the influence of particle size on burnability without introducing excessive grinding effort or compromising material stability. While broader or finer ranges could be explored in future studies, the chosen interval was sufficient to observe meaningful differences among raw materials under practical conditions.

**Figure 3:**
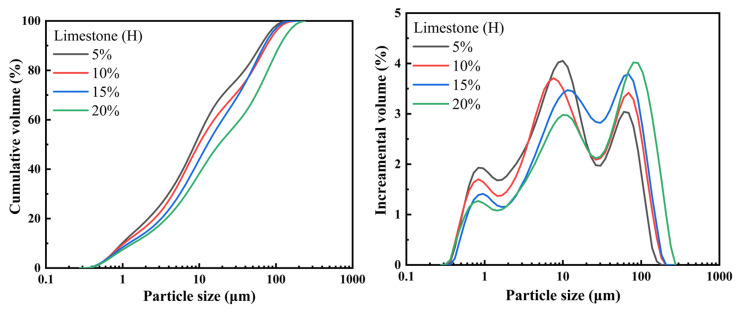

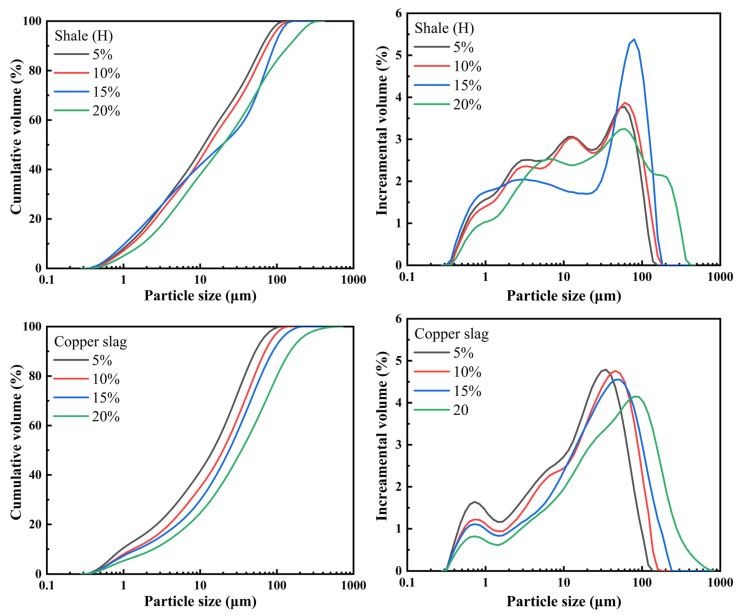
PSD of ground raw materials.

Ground samples were sealed in plastic bags and stored prior to use. Particle size distributions (PSD) at each fineness level were measured using a laser diffraction analyzer (90PlusPALS, Brookhaven, Nashua, NH, USA), while specific surface areas were determined via nitrogen adsorption. All samples were oven-dried for 10 h before analysis. The PSD profiles of the ground H-type raw materials are shown in Figure 3. The corresponding D10, D50, D90 values and average particle sizes for each fineness level are summarized in Table 5.

#### 2.2.3. Preparation of Cement Clinker

Based on the proportioning schemes outlined in Table 3, the raw materials were blended in a V-type mixer for 10 min, then compacted into cylindrical pellets (Φ17 × 13 mm^3^) using a steel mold. The pellets were placed in crucibles and subjected to thermal processing in a high-temperature electric furnace, with the heating rate controlled at 10 °C/min. The samples were first preheated at 950 °C for 30 min to ensure complete decomposition of calcium carbonate, then sintered at 1450 °C for another 30 min to facilitate clinker formation. After firing, the clinkers were rapidly cooled to approximately 25 °C using forced air.

#### 2.2.4. Preparation of Cement Paste and Mortar

Following Chinese National Standard GB 175-2023, Portland cement was produced by blending 95 wt.% ground clinker with 5 wt.% calcium sulfate dihydrate (gypsum) in a V-type mixer for 10 min. The standard consistency water demand and setting times of the cement paste were determined following GB/T 1346-2011. Cement mortar was prepared as per the guidelines in GB/T 17671-2021. 

Compressive and flexural strength tests were separately performed using an electronic universal testing machine (CDT1305-2, Mesiste Industrial Co., Ltd., China). Each strength value represents the average of three mortar specimens. For hydration analysis, cement paste samples cured for 28 days were crushed, immersed in absolute ethanol for 72 h to halt hydration, and then dried in a vacuum oven at 50 °C.

## 3. Structure and Discussion

### 3.1. Influence of Raw Material Particle Size Distribution on the Burnability of Raw Meal

#### 3.1.1. Burnability Test of Raw Meal

Burnability tests of cement raw meal were carried out following the Chinese National Standard GB/T 26566-2011. Limestone, shale, and iron-bearing corrective materials were individually ground to obtain a 10% residue on an 80 μm sieve, resulting in a raw meal with a similar 10% residue at the same sieve size. Following pre-calcination at 950 °C for 30 min, the raw meal samples were calcined for 30 min at three different temperatures: 1350 °C, 1400 °C, and 1450 °C. After calcination, the clinkers were air-cooled to room temperature.

The f-CaO content in the clinkers was determined using the glycerol–ethanol method outlined in GB/T 176-2017. The results are presented in Table 6.

As illustrated in Table 6, the f-CaO content in the clinker gradually declines as the calcination temperature rises. At 1450 °C, the f-CaO content in both X and H clinkers falls below 1.5 wt.%, indicating complete calcination under these conditions. These values meet the requirements specified in the Chinese National Standard GB/T 21372-2024.

#### 3.1.2. Influence of Fineness of Silica-Alumina Raw Materials on the Burnability of Raw Meal

**Figure 4:**
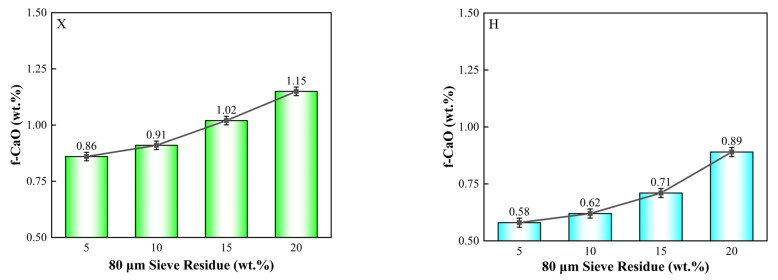
Comparison of burnability of raw meal with varying fineness of silica-alumina raw materials.

The fineness of limestone and iron-bearing corrective materials was maintained with a residue of 10% on the 80 μm sieve. The effect of the fineness of silica-alumina raw materials on the burnability of the raw meal was investigated by varying their particle size. The calcination temperature was set at 1450 °C. The specific proportioning scheme is provided in Table 7, and the experimental results are presented in Figure 4.

As shown in Figure 4, within the residue range of 5% to 20%, the impact of shale fineness on the burnability of the raw meal is limited. As the shale residue increases, the burnability of the raw meal slightly decreases. At a residue of 5%, the f-CaO content is 0.86% for X and 0.58% for H. When the residue increases to 20%, the f-CaO content rises to 1.15% for X and 0.89% for H.

Further comparison reveals that, as the shale residue increases from 5% to 20%, the f-CaO content increases by 0.29% in the X group (with a shale mass fraction of 8.09%) and by 0.31% in the H group (with a shale mass fraction of 13.37%). The increase in both cases is small, indicating that, within this range of fineness, the effect of shale fineness on the burnability of the raw meal is minor and can be considered a secondary factor.

#### 3.1.3. Influence of Fineness of Iron-Bearing Corrective Materials on the Burnability of Raw Meal

**Figure 5:**
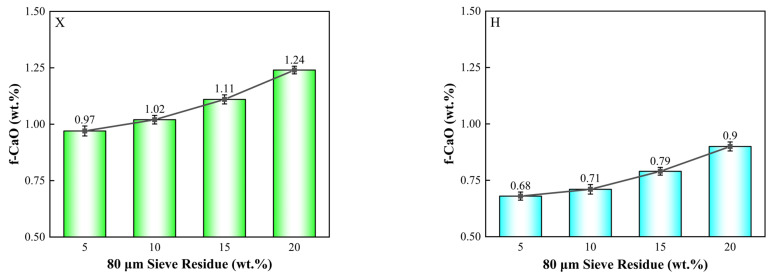
Comparison of burnability of raw meal with varying fineness of iron-bearing corrective materials.

Based on the conclusions from Section 3.1.2, it was observed that when the shale residue increased from 10% to 15%, the f-CaO content in the clinker changed by less than 0.2%. Therefore, shale with a residue of 15% was selected for subsequent experiments. The fineness of limestone and shale was kept constant, while the fineness of iron tailings and copper slag was varied to examine the effect of the particle size of iron-bearing corrective materials on the burnability of the raw meal. The experimental proportioning scheme is outlined in Table 8, and the results are presented in Figure 5.

As shown in Figure 5, within the residue range of 5% to 20%, the effect of the fineness of iron-bearing corrective materials on the burnability of the raw meal is limited. The experimental data indicate that, as the residue increases from 5% to 20%, the f-CaO content in X clinker rises from 0.97% to 1.24%, an increase of 0.27%. In H clinker, f-CaO increases from 0.68% to 0.90%, an increase of 0.22%. Thus, within this fineness range, the particle size of iron-bearing corrective materials has a minor effect on the burnability of the raw meal and can be considered a secondary factor in the context of this study.

#### 3.1.4. Influence of Fineness of Calcareous Raw Materials on the Burnability of Raw Meal

**Figure 6:**
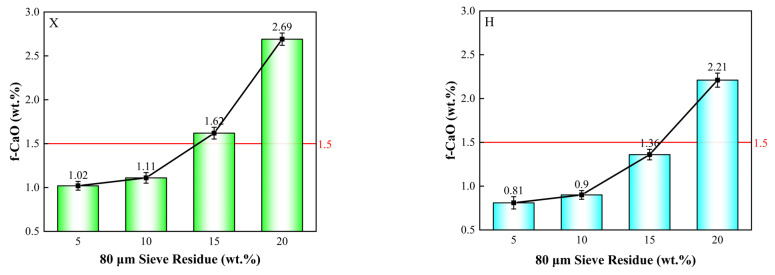
Comparison of burnability of raw meal with varying fineness of calcareous raw materials.

Based on the findings from Section 3.1.3, when the residue of iron tailings in X increased from 10% to 15%, and the residue of copper slag in H increased from 10% to 20%, the change in f-CaO content was less than 0.2%. Therefore, iron tailings with a residue of 15% and copper slag with a residue of 20% were selected for subsequent experiments. The fineness of shale and iron-bearing corrective materials was kept constant, while the fineness of limestone was varied to study its impact on the burnability of the raw meal. The experimental proportioning scheme is outlined in Table 9, and the results are presented in Figure 6.

As shown in Figure 6, the fineness of limestone significantly affects the burnability of the raw meal. As the primary source of CaO, limestone constitutes more than 80% of the raw meal. The experimental results show that, as the residue of limestone increases from 5% to 20%, the f-CaO content in X clinker rises from 1.02% to 2.69%, and in H clinker, from 0.81% to 2.21%. This suggests that a higher limestone residue leads to an increase in f-CaO content in the clinker, indicating incomplete reaction of CaO. Therefore, optimizing the fineness of limestone is a crucial method for improving the burnability of the raw meal.

Since the f-CaO content in X clinker with a limestone residue of 15% is 1.62%, which exceeds the 1.5% upper limit set by the Chinese National Standard GB/T 21372-2024, additional experiments were conducted with limestone residues of 13% and 14%. The results are presented in Table 10.

The experimental results demonstrate that when the limestone residue in the X group is 13%, the f-CaO content of the clinker meets the national standard.

The raw meal burnability tests indicate that finer control over PSD facilitates clinker phase formation. This may allow for a reduction in sintering temperature, thereby lowering fuel consumption and CO_2_ emissions during clinker production. However, achieving such benefits requires avoiding overly coarse raw meal preparation. While direct quantification of energy savings was beyond the scope of this study, the results suggest that PSD optimization could serve as a viable strategy to improve thermal efficiency and environmental performance in cement manufacturing.

### 3.2. Comparison of Chemical and Mineralogical Characteristics of Clinker Before and After Formula Optimization

**Figure 7:**
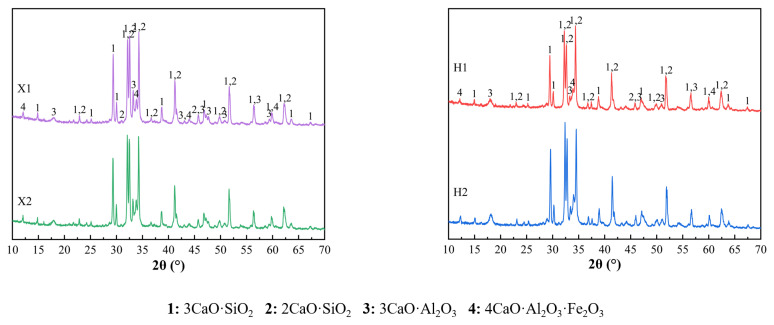
XRD analysis of X1, X2, and H1, H2 clinkers.

Based on the findings from Section 3.1, when the residue of limestone, shale, and iron tailings in X raw meal is 13%, 15%, and 15%, respectively, and in H raw meal, the residue of limestone, shale, and iron tailings is 15%, 15%, and 20%, the f-CaO content in the clinker remains below 1.5%. Therefore, following this fineness optimization scheme, two sets of comparative experiments were designed: one with coarse and fine raw meal and the other with a finer raw meal. These experiments aim to explore the impact of comprehensive raw material particle size control on clinker firing characteristics. The specific raw meal proportioning scheme is outlined in Table 11, and the XRD results are presented in Figure 7.

From the XRD analysis results of X1, X2, H1, and H2 clinkers shown in Figure 7, it is evident that the diffraction peaks of the mineral phases C_3_S, C_2_S, C_3_A, and C_4_AF are consistent in both peak position and intensity, indicating no significant change in the major mineral composition. Although X1 and X2, as well as H1 and H2, correspond to raw materials with different particle sizes, the diffraction peak positions and relative intensities of mineral phases in each sample group are largely consistent. This suggests that, under identical firing conditions, the mineral composition of the clinker remains stable, predominantly consisting of C_3_S, C_2_S, C_3_A, and C_4_AF.

Further comparison of the XRD patterns of X1 vs. X2 and H1 vs. H2 reveals that the characteristic diffraction peaks of C_3_S and C_2_S consistently appear at their typical 2θ positions, with minimal differences in peak shape and intensity. Similarly, the characteristic peaks of C_3_A and C_4_AF maintain high consistency, indicating that the mineral phases of raw meals with different particle sizes are essentially the same under the same firing conditions [28].

In conclusion, within the residue range of 10–15%, variations in raw meal fineness have a minimal effect on the major mineral composition of the clinker. The differences in raw meal particle size within this range have a limited impact on the formation of key mineral phases, thereby maintaining the consistency of clinker composition.

### 3.3. Comparison of Cement Performance and Hydration Products Before and After Formulation Optimization

Changes in raw material fineness not only affect the burnability of clinker but may also influence cement performance. To comprehensively assess the impact of the proposed fineness optimization on cement quality, it is essential to further investigate its effects on the properties of cement mortar and paste, ensuring the stability of the final product’s performance.

Therefore, in this section, cement is prepared using both the pre-optimization raw material fineness scheme (X1, H1) and the post-optimization scheme (X2, H2). The mechanical properties and hydration products are tested to evaluate the effect of raw material fineness optimization on cement performance.

#### 3.3.1. Comparison of Cement Performance

The setting time, standard consistency, and stability of the four cement samples were tested, with the results summarized in Table 12.

As shown in Table 12, the setting time, standard consistency, soundness, and specific surface area of all six cement samples meet the requirements specified in the Chinese National Standard for Portland cement (GB 175-2023). Furthermore, the difference in standard consistency between X2 and X1, as well as between H2 and H1, is less than 1.5%, and the changes in initial and final setting times remain within 10 min. This indicates that the adjustments in fineness did not cause any significant performance fluctuations [29].

**Figure 8:**
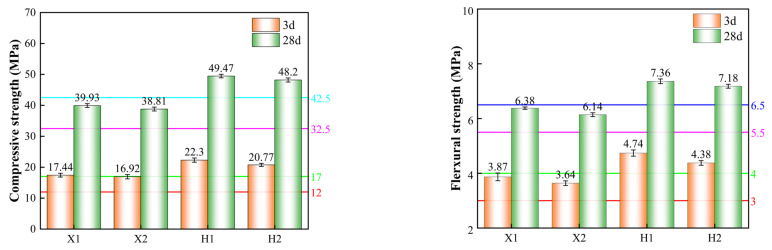
Compressive and flexural strengths of cement mortar.

The compressive and flexural strengths of the cement mortar after 28 days of curing were measured, and the results are presented in Figure 8.

As shown in Figure 8, the 3-day flexural and compressive strengths of the H1 and H2 cement samples were 4.74 MPa and 4.38 MPa, and 22.3 MPa and 20.77 MPa, respectively. The 28-day flexural and compressive strengths were 7.36 MPa and 7.18 MPa, and 49.47 MPa and 48.2 MPa, respectively, meeting the requirements for a 42.5 grade cement. The 3-day flexural and compressive strengths of the X1 and X2 cement samples were 3.87 MPa and 3.64 MPa, and 17.44 MPa and 16.92 MPa, respectively, while the 28-day flexural and compressive strengths were 6.38 MPa and 6.14 MPa, and 39.93 MPa and 38.81 MPa, respectively, meeting the requirements for a 32.5 grade cement.

These results suggest that the raw material fineness optimization scheme maintains stable mechanical properties without significantly altering the cement’s setting time or standard consistency, indicating that the optimization does not adversely affect cement performance.

#### 3.3.2. XRD Comparison of Cement Paste

**Figure 9:**
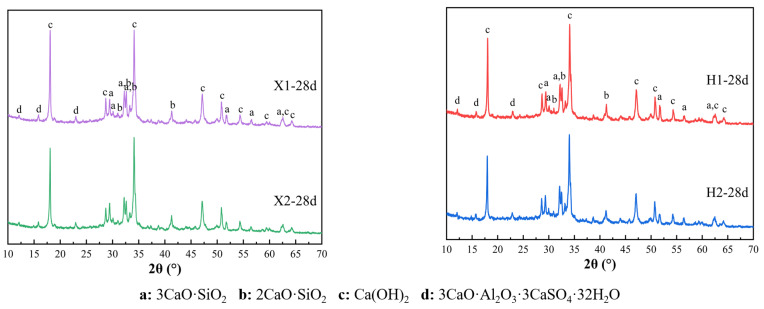
XRD of the 28-day hydration products of X1, X2, H1, and H2.

Cement paste was prepared, and XRD analysis was conducted on the hydration products after 28 days of curing, as shown in Figure 9.

Analysis of the XRD patterns of the X1, X2, H1, and H2 samples at 28 days of curing reveals the presence of calcium hydroxide (Ca(OH)_2_) and ettringite (AFt) in all samples, while certain amounts of unhydrated tricalcium silicate (C_3_S) and dicalcium silicate (C_2_S) remained. This indicates that the hydration product composition is consistent across all samples, with similar levels of unreacted materials.

Comparison of the intensity of the C_3_S and C_2_S diffraction peaks among the four groups of samples shows minimal variation in peak intensity, suggesting that the hydration degree of the cement prepared under different raw material fineness conditions is essentially the same at this age. The variation in raw material fineness within the 10–15% sieve residue range does not significantly affect the residual unhydrated minerals. Ca(OH)_2_, the primary alkaline product of C_3_S and C_2_S hydration, exhibits clear diffraction peaks in all samples with consistent peak intensities, indicating similar levels of hydration reaction [30]. AFt is also present in all samples, with comparable peak intensities, suggesting that its formation is similar across the samples [31].

In conclusion, the differences in the types and relative contents of hydration products at 28 days are minimal among the four cement samples, indicating that variations in raw material fineness within the 10–15% sieve residue range do not significantly influence the cement’s hydration behavior.

#### 3.3.3. TG-DTG Comparison of Cement Paste

**Figure 10:**
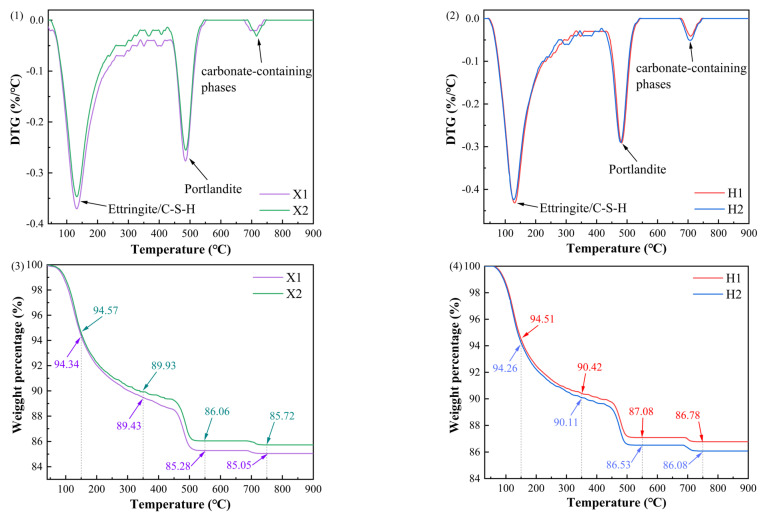
TG-DTG curves of the hydration products of cement paste cured for 28 days.

Figure 10 presents the TG-DTG curves of the hydration products of cement paste cured for 28 days. As shown in Figure 10(1,2), the DTG curves of H1, H2, X1, and X2 display two major peaks and one minor peak. The weight loss observed between approximately 50 °C and 200 °C corresponds to AFt and C-S-H gel. The peak between 450 °C and 550 °C is associated with the decomposition of calcium hydroxide (Ca(OH)_2_) [32], leading to the release of water and the formation of CaO. The minor peak between 680 °C and 750 °C is attributed to the decomposition of carbonate phases [33], primarily resulting from the reaction of hydration products with atmospheric CO_2_.

Decomposition peaks of ettringite, C-S-H gel, and calcium hydroxide, along with the presence of carbonate phases, are observed in all samples. According to studies by Fang and Chang [34], and Šavija and Luković [35], the carbonation of ettringite, C-S-H gel, and calcium hydroxide accounts for 70–80% of the total carbonation, while the remaining carbonation could be attributed to partial carbonation of the hydrated clinker or other byproducts.

As shown in Figure 10(3,4), the TG curves indicate that at 900 °C, the mass loss of X1 and X2 is 13.94% and 14.72%, respectively, while for H1 and H2, it is 13.22% and 13.92%. The difference does not exceed 0.8%, suggesting that the hydration degree and thermal stability of the samples are consistent.

Therefore, the types and decomposition behavior of the hydration products of X2 and X1, as well as H2 and H1, are essentially identical, indicating that variations in raw material fineness within the 10–15% sieve residue range do not significantly affect the cement’s hydration reaction. The decomposition peak of Portlandite between 400–500 °C is nearly identical for all samples, supporting the conclusion that the cement samples exhibit similar hydration degrees at 28 days, with no significant differences caused by changes in raw material fineness. The mass loss data suggest that the quantity of hydration products for the slightly coarser cement is comparable to that of the finer cement, indicating that the basic performance of the cement is unaffected.

These findings suggest that a differentiated grinding strategy could be implemented in cement plants—specifically, applying finer grinding to limestone while allowing coarser grinding of shale and iron-bearing materials. Such an approach could optimize energy consumption during raw material preparation without compromising clinker quality or cement performance.

## 4. Conclusions

An analysis was conducted on the burnability of clinker prepared from raw materials with varying fineness, including limestone, shale, and iron corrective materials. Additionally, a comparison and performance testing of cement prepared from coarser and finer raw materials were carried out. The following conclusions were drawn:

Effect of Raw Material Fineness on Clinker Burnability: The influence of different raw materials on the burnability of the clinker varies. Within the range of 5–20% sieve residue at 80 μm, variations in the fineness of silicate-aluminate and iron corrective materials have a minimal impact on burnability, indicating that these are not the primary factors. In contrast, the fineness of calcareous raw materials significantly affects the f-CaO content, making it a key factor controlling the burnability of clinker.

Impact of Fineness on Mineral Composition: Changes in raw material fineness have a limited impact on the major mineral composition of the clinker. A comparison of XRD patterns for clinker produced from raw materials with different fineness shows that, within the sieve residue range of 10–15%, the content of major minerals in the clinker remains largely unchanged. This suggests that fluctuations in fineness within this range do not significantly influence mineral formation.

Adaptability of Cement Performance to Raw Material Fineness: Cement performance demonstrates strong adaptability to variations in raw material fineness. Cement prepared from clinker produced at different fineness levels was subjected to mechanical property testing and hydration product analysis. The results indicate that, within the aforementioned range of fineness variation, no significant differences were observed in terms of strength, hydration product types, or relative content. This suggests that a moderate increase in sieve residue of raw materials does not significantly impact the final performance of the cement. The results support the feasibility of a selective grinding strategy to improve energy efficiency while maintaining overall cement quality.

## Figures and Tables

**Table 1:** Chemical composition of cement raw materials measured by XRF (wt.%).

Raw Material Name	CaO	SiO_2_	Al_2_O_3_	Fe_2_O_3_	LOI ^1^	MgO	SO_3_	K_2_O	Na_2_O
Limestone (X)	48.22	7.18	2.65	0.54	40.34	0.77	0.12	0.2	0.09
Shale (X)	0.83	67.54	14.47	6.43	4.9	1.2	0.05	2.99	0.44
Iron Tailings	14.38	45.12	4.06	22.64	9.84	3.27	0.22	0.47	0.16
Gypsum	48.51	---	---	---	20.12	---	31.21	---	---
Limestone (H)	51.19	5.58	0.66	0.26	40.15	0.78	0.06	0.06	0.06
Shale (H)	0.73	62.74	14.83	8.35	6.16	2.77	0.32	2.06	0.31
Copper Slag	2.96	30.97	3.23	58.49	0	1.85	0.12	0.54	0.33

^1^ LOI refers to the percentage of mass loss upon heating to 950 °C.

**Table 2:** Hardness and density of cement raw materials.

Raw Material	Limestone (X)	Shale (X)	Iron Tailings	Limestone (H)	Shale (H)	Copper Slag
Density (g/cm^3^)	2.59	2.51	2.85	2.61	2.54	3.66

**Table 3:** Raw meal proportioning schemes for cement production.

	Raw Material Proportion (wt%)			KH ^1^	SM ^2^	IM ^3^
X	Limestone (X)	Shale (X)	Iron Tailings	0.9	2.39	1.65
	86.4	8.09	5.5			
H	Limestone (H)	Shale (H)	Copper Slag	0.9	2.5	1.39
	84.84	13.37	1.79			

^1^ KH = $\frac{\omega (\mathrm{CaO})-1.65\cdot {\omega (\mathrm{Al}}_{2}O_{3})-0.35\cdot {\omega (\mathrm{Fe}}_{2}O_{3})}{2.8\cdot {\omega (\mathrm{SiO}}_{2})}$. ^2^ SM = $\frac{{\omega (\mathrm{SiO}}_{2})}{{\omega (\mathrm{Al}}_{2}O_{3}{)+\omega (\mathrm{Fe}}_{2}O_{3})}$. ^3^ IM = $\frac{{\omega (\mathrm{Al}}_{2}O_{3})}{{\omega (\mathrm{Fe}}_{2}O_{3})}$.

**Table 4:** Fineness of different raw materials.

Limestone (X)		Limestone (H)		Shale (X)		Shale (H)		Copper Slag		Iron Tailings	
Sample ID	Sieve Residue (%)	Sample ID	Sieve Residue (%)	Sample ID	Sieve Residue (%)	Sample ID	Sieve Residue (%)	Sample ID	Sieve Residue (%)	Sample ID	Sieve Residue (%)
XS-1	5	HS-1	5	XY-1	5	HY-1	5	XT-1	5	HT-1	5
XS-2	10	HS-2	10	XY-2	10	HY-2	10	XT-2	10	HT-2	10
XS-3	15	HS-3	15	XY-3	15	HY-3	15	XT-3	15	HT-3	15
XS-4	20	HS-4	20	XY-4	20	HY-4	20	XT-4	20	HT-4	20

**Table 5:** PSD of raw materials.

	D10% (μm)	D50% (μm)	D90% (μm)
HS-5%	0.983	8.772	63.179
HS-10%	1.043	9.844	75.232
HS-15%	1.198	12.77	84.154
HS-20%	1.373	18.069	112.184
HY-5%	1.174	11.183	64.241
HY-10%	1.296	13.097	74.438
HY-15%	1.032	19.363	92.726
HY-20%	1.767	19.863	143.182
HT-5%	0.95	14.644	52.428
HT-10%	1.327	19.877	70.038
HT-15%	1.535	23.907	88.528
HT-20%	2.455	34.687	149.078

**Table 6:** f-CaO content in cement clinker.

	1350 °C	1400 °C	1450 °C
X	3.12 wt.%	1.84 wt.%	0.98 wt.%
H	2.38 wt.%	1.16 wt.%	0.62 wt.%

**Table 7:** Burnability proportioning scheme with varying fineness of silica-alumina raw materials.

Sample ID	Limestone			Shale			Iron Tailings or Copper Slag			Raw Meal Fineness (%)
	Raw Material ID	Sieve Residue (%)	Blend Ratio (%)	Raw Material ID	Sieve Residue (%)	Blend Ratio (%)	Raw Material ID	Sieve Residue (%)	Blend Ratio (%)	
X-01	XS-2	10	86.4	XY-1	5	8.09	XT-2	10	5.51	9.60
X-02	XS-2	10	86.4	XY-2	10	8.09	XT-2	10	5.51	10.00
X-03	XS-2	10	86.4	XY-3	15	8.09	XT-2	10	5.51	10.40
X-04	XS-2	10	86.4	XY-4	20	8.09	XT-2	10	5.51	10.81
H-01	HS-2	10	84.84	HY-1	5	13.37	HT-2	10	1.79	9.33
H-02	HS-2	10	84.84	HY-2	10	13.37	HT-2	10	1.79	10.00
H-03	HS-2	10	84.84	HY-3	15	13.37	HT-2	10	1.79	10.67
H-04	HS-2	10	84.84	HY-4	20	13.37	HT-2	10	1.79	11.34

**Table 8:** Burnability proportioning scheme with varying fineness of iron-bearing corrective materials.

Sample ID	Limestone			Shale			Iron Tailings or Copper Slag			Raw Meal Fineness (%)
	Raw Material ID	Sieve Residue (%)	Blend Ratio (%)	Raw Material ID	Sieve Residue (%)	Blend Ratio (%)	Raw Material ID	Sieve Residue (%)	Blend Ratio (%)	
X-05	XS-2	10	86.4	XY-3	15	8.09	XT-1	5	5.51	10.13
X-06	XS-2	10	86.4	XY-3	15	8.09	XT-2	10	5.51	10.40
X-07	XS-2	10	86.4	XY-3	15	8.09	XT-3	15	5.51	10.68
X-08	XS-2	10	86.4	XY-3	15	8.09	XT-4	20	5.51	10.96
H-05	HS-2	10	84.84	HY-3	15	13.37	HT-1	5	1.79	10.58
H-06	HS-2	10	84.84	HY-3	15	13.37	HT-2	10	1.79	10.67
H-07	HS-2	10	84.84	HY-3	15	13.37	HT-3	15	1.79	10.76
H-08	HS-2	10	84.84	HY-3	15	13.37	HT-4	20	1.79	10.85

**Table 9:** Burnability proportioning scheme with varying fineness of calcareous raw materials.

Sample ID	Limestone			Shale			Iron Tailings or Copper Slag			Raw Meal Fineness (%)
	Raw Material ID	Sieve Residue (%)	Blend Ratio (%)	Raw Material ID	Sieve Residue (%)	Blend Ratio (%)	Raw Material ID	Sieve Residue (%)	Blend Ratio (%)	
X-09	XS-1	5	86.4	XY-3	15	8.09	XT-4	15	5.51	6.36
X-10	XS-2	10	86.4	XY-3	15	8.09	XT-4	15	5.51	10.68
X-11	XS-3	15	86.4	XY-3	15	8.09	XT-4	15	5.51	15.00
X-12	XS-4	20	86.4	XY-3	15	8.09	XT-4	15	5.51	19.32
H-09	HS-1	5	84.84	HY-3	15	13.37	HT-4	20	1.79	6.61
H-10	HS-2	10	84.84	HY-3	15	13.37	HT-4	20	1.79	10.85
H-11	HS-3	15	84.84	HY-3	15	13.37	HT-4	20	1.79	15.09
H-12	HS-4	20	84.84	HY-3	15	13.37	HT-4	20	1.79	19.33

**Table 10:** f-CaO content of X group with limestone residues of 13% and 14%.

sieve residue (%)	13	14
f-CaO (wt.%)	1.39	1.51

**Table 11:** Comparison analysis scheme of coarse and fine raw meal.

Sample ID	Limestone		Shale		Iron Tailings or Copper Slag		Raw Meal Fineness (%)
	Sieve Residue (%)	Blend Ratio (%)	Sieve Residue (%)	Blend Ratio (%)	Sieve Residue (%)	Blend Ratio (%)	
X1	10	86.4	10	8.09	10	5.51	10
X2	13	86.4	15	8.09	15	5.51	13.27
H1	10	84.84	10	13.37	10	1.79	10
H2	15	84.84	15	13.37	20	1.79	15.09

**Table 12:** Setting time and standard consistency of cement.

Sample ID	Solidification Time (min)		Standard Consistency (%)	Stability (mm)	Specific Surface Area (m^2^/kg)
	Initial Condensation	Complete Condensation			
X0	158	249	25.6	1.5	337
X1	164	263	26.9	1.9	331
H0	122	225	24.1	0.8	343
H1	127	232	24.9	1.5	336
Standard	≥45	≤390	---	≤5	300–400

## Data Availability

The original contributions presented in this study are included in the article. Further inquiries can be directed to the corresponding author.
